# Niche partitioning between sympatric wild canids: the case of the golden jackal (*Canis aureus*) and the red fox (*Vulpes vulpes*) in north-eastern Italy

**DOI:** 10.1186/s12862-021-01860-3

**Published:** 2021-06-22

**Authors:** Elisa Torretta, Luca Riboldi, Elena Costa, Claudio Delfoco, Erica Frignani, Alberto Meriggi

**Affiliations:** 1 Department of Earth and Environmental Sciences, University of Pavia, Via Ferrata 1, 27100 Pavia, Italy; 2 Department of Chemistry, Life Sciences and Environmental Sustainability, University of Parma, Parco Area delle Scienze 11/a, 43124 Parma, Italy

**Keywords:** Competition, Interference, Niche overlap, Niche partitioning, Camera trapping, Scat analysis, Utilization distributions, Activity patterns, Ecological Niche Factor Analysis (ENFA), Diet

## Abstract

**Background:**

Two coexisting species with similar ecological requirements avoid or reduce competition by changing the extent of their use of a given resource. Numerous coexistence mechanisms have been proposed, but species interactions can also be aggressive; thus, generally a subordinate species modifies its realized niche to limit the probability of direct encounters with the dominant species. We studied niche partitioning between two sympatric wild canids in north-eastern Italy: the golden jackal and the red fox, which, based on competition theories, have a high potential for competition. We considered four main niche dimensions: space, habitat, time, and diet.

**Results:**

We investigated three study areas monitoring target species populations from March 2017 to November 2018 using non-invasive monitoring techniques. Red fox presence was ascertained in every study area, while golden jackal presence was not ascertained in one study area, where we collected data regarding wolf presence. Considering the two target species, we observed partial diet partitioning based on prey size, with the golden jackal mainly feeding on wild ungulates and the red fox mainly feeding on small mammals. The two canids had an extensive temporal overlap along the diel cycle, having both predominant crepuscular and nocturnal activity patterns, but marked spatial partitioning and differential use of habitats. The golden jackal proved to be specialist concerning the habitat dimension, while the red fox resulted completely generalist: the former selected less human-modified habitats and avoided intensively cultivated lands, while the latter was present in all habitats, including intensively cultivated lands.

**Conclusions:**

The observed partitioning might be due partially to some ecological adaptations (e.g. specialist vs. generalist use of resources) and specific behaviours (e.g. cooperative vs. solitary hunting) and partially to the avoidance response of the red fox aimed at reducing the probability of direct encounters with the golden jackal.

**Supplementary Information:**

The online version contains supplementary material available at 10.1186/s12862-021-01860-3.

## Introduction

Sympatric species with similar ecological requirements can either coexist or competitively exclude each other depending on resources availability: the strength of the competition between them generally decreases with increased differentiated resources use ([1] and references therein). Considering carnivores, exploitation [2] and interference [3] have been identified as key mechanisms structuring the guild; the magnitude of interspecific aggressive behaviours is generally driven by relative differences in body sizes (i.e. aggressive behaviours are more frequent when the body mass ratio of the contenders ranges between 2 and 5.4), dietary overlap, predatory habits, and taxonomic similarity (i.e. aggressive behaviours are more frequent between species of the same family) [4, 5]. Because these interactions are generally asymmetric (subordinate vs. dominant), generally the subordinate species modify its realized niches by changing the extent of its use of resources.

Competitive interactions among wild canids have been frequently recorded [3, 6] and have been widely investigated. In North America, for example, many researchers focused on the cascading interactions involving the wolf (*Canis lupus*), the coyote (*Canis latrans*), and foxes (*Vulpes sp.* and *Urocyon cinereoargentus*) ([7] and references therein, [8]). In Africa the complexity of the carnivores’ guild promoted substantial prior researches focused on the interactions involving different wild canids, as the spotted hyena (*Crocuta crocuta*) and the African wild dog (*Lycaon pictus*), and other larger species, the lion (*Panthera leo*) above all (e.g. [9–12]). Regarding Eurasia, many studies dealing with canids interactions focused on a single (or a few) niche dimension ([13–17], but see [18]).

Overall, two coexisting species with similar ecological requirements limit interspecific overlap by changing the extent of their use of a given resource to avoid or reduce competition. This process is known as niche partitioning [19]. Numerous coexistence mechanisms have been proposed, including spatial segregation, variations in habitat use, behavioural adaptations and altered activity periods or movements, trophic segregation and specialization [20].

To gain a significant understanding of the coexistence mechanisms between potentially competing species, more than a single dimension associated with an ecological niche should be considered [21, 22]. Therefore, we investigated multi-dimension niche partitioning between two sympatric wild canids: the golden jackal (*Canis aureus*) and the red fox (*Vulpes vulpes*). The golden jackal (7–15 kg) is a widespread species throughout southern Asia, the Middle East and south-eastern and central Europe [23], where it inhabits a wide variety of habitats in different bioclimatic areas: from semi-deserts and grasslands to forests, but also agricultural and semi-urban habitats [24–27]. The red fox (4–11 kg), being the most common mesocarnivore in the northern hemisphere, is widely distributed [28]; it is trophic and habitat generalist known for its opportunistic behaviour and adaptability to human-dominated landscapes [29–31]. Evidence of competition between the two species have been occasionally recorded: in Israel, for example, in areas where golden jackals became very abundant, the population size of red foxes decreased significantly, apparently because of exclusion by golden jackals (reported in [32]). Moreover, more recently, a mechanism of spatial segregation has been documented, in which red foxes avoided the core activity areas of golden jackals and restricted their activity to their peripheries [33].

These canids are sympatric in north-eastern Italy since the expansion of the golden jackal in this area started in 1984 [34]. We carried out an integrated research in which we considered golden jackal and red fox resource partitioning along different niche dimensions: (i) space, (ii) habitat, (iii) time, and (iv) diet. Considering multiple dimensions associated with an ecological niche is mandatory because partitioning and overlap along the main niche dimensions are counterbalancing; in other words, similarities along one dimension should imply dissimilarities along another one [19]. For each dimension, we evaluated golden jackal and red fox use of resources and their degree of overlap.

According to the theories concerning the interference interactions and mechanisms of co-existence among carnivores [4, 6, 35] and the few evidences [33, 32] regarding the competition between these two species, we hypothesised that the coexistence between the golden jackal and the red fox would be favoured by the differentiation along one or more niche axes, which would relieve interspecific potential competition and facilitate coexistence between them, though their similar ecological requirements. We expected at least a partial partitioning between the two species at the spatial dimension because spatial segregation has already been documented between them [33, 32]. Further, we expected a partial partitioning at the trophic dimension, because of the differences in their body size [36]. Potentially connected with these dimensions, we expected also a partial partitioning along habitat dimension. Finally, in case of no partitioning over these dimensions (especially spatial and trophic), we predicted that temporal partitioning would have played a significant role in separating their ecological niches.

## Methods

### Study areas

**Fig. 1:**
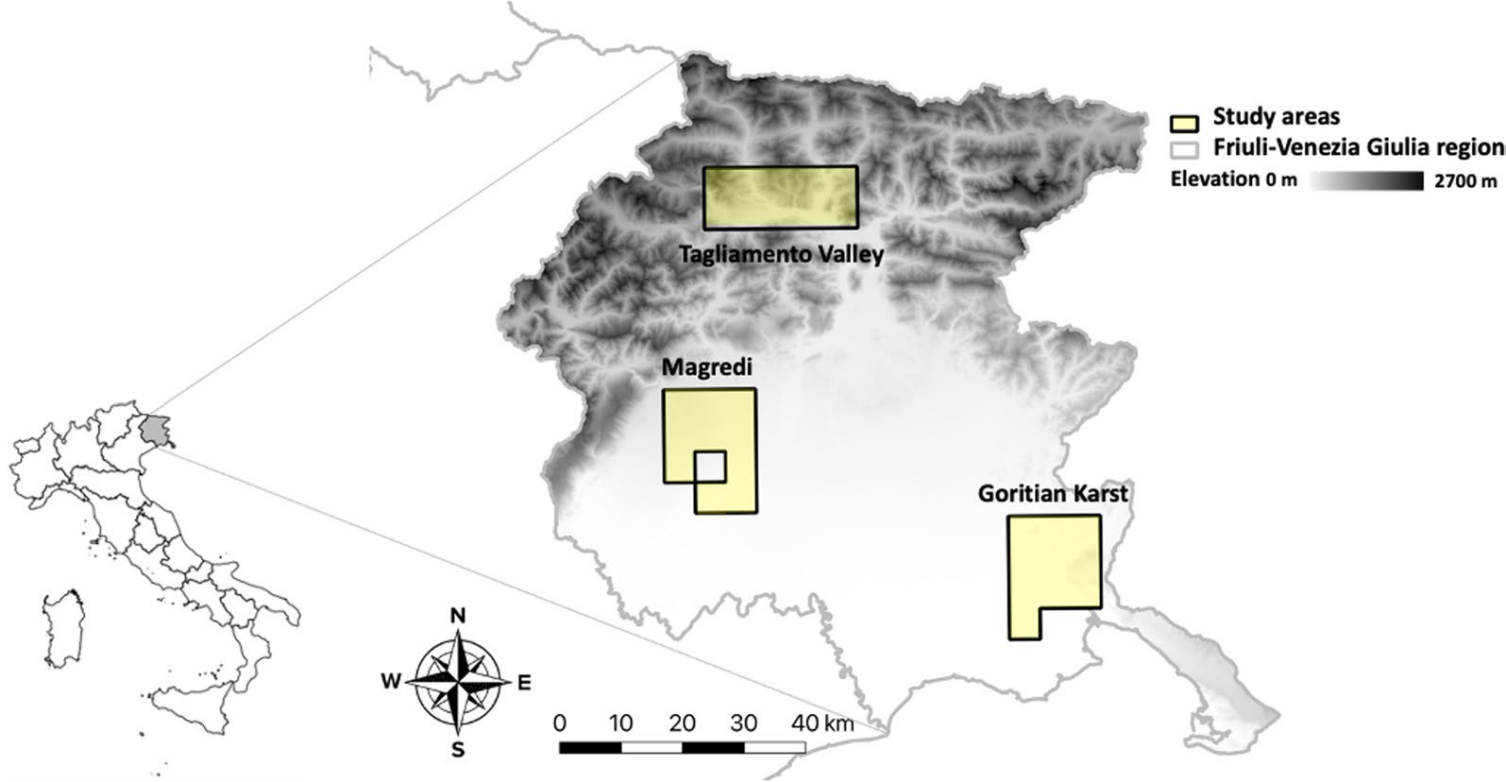
Location of the three study areas in Friuli–Venezia Giulia region

This research was carried out in Friuli–Venezia Giulia region (north-eastern Italy), where we sampled three study areas covering a total surface of 750 km^2^ (Fig. 1). The Goritian Karst is located in the south-eastern part of the region and includes the Karst plateau and the surrounding intensively cultivated plain. The Magredi area is located in the western part of the region and it is characterized by the presence of subterranean rivers surrounded by an intensively cultivated landscape. Finally, the Tagliamento Valley is a typical mountain area located in the northern part of the region in the Alps (Table 1). The presence of both the golden jackal and the red fox was ascertained in every study area before the beginning of this research [37–40]. In particular, golden jackal reproduction was confirmed in the Karst since the early 1990s, while it was documented more recently (2010) in the Tagliamento Valley and the Magredi area [40]. According to the National Law 157/92, golden jackal hunting was forbidden, but red fox hunting was allowed in autumn–winter. Further details on the study areas are provided in Torretta et al. [41].

**Table 1:** Main characteristics of the study areas

	Goritian Karst	Magredi	Tagliamento Valley
	13°27′37'' N; 45°51′39'' E	12°45′23'' N; 46°04′11'' E	12°55′50'' N; 46°25′27'' E
Area (km^2^)	250	250	250
Elevation (m a.s.l.)	2–225	32–311	293–1954
Land cover			
Urban areas	14.0%	4.1%	3.2%
Agricultural areas:			
Intensively cultivated lands	46.4% [mainly cereals and legumes]	42.1% [mainly cereals and legumes]	
Permanent crops	3.5% [mainly vineyards]	5.9% [mainly vineyards]	
Complex cultivation patterns	8.1%	19.3%	1.3%
Agricultural areas with natural vegetation	6.2%	1.6%	8.8%
Woodlands:			
Broad-leaved woodlands	5.6%	0.1%	22.0%
Coniferous woodlands	2.2%		4.5%
Mixed woodlands	2.4%		41.2%
Meadows and pastures	0.9%	4.1%	6.6%
Shrublands	7.9%	1.3%	2.9%
River banks without vegetation	2.0%	9.7%	4.3%
Sparse vegetation		11.8%	5.1%
Bare rocks			0.2%
Water bodies	0.9%		
Climate	Continental	Continental	Alpine
Mean annual temperature (°C)	13.3	13.2	10.0
Mean annual rainfall (mm)	1200–1800	1200–1800	2700–3200
Mammals (potential prey)			
Wild ungulates	*Capreolus capreolus* *Cervus elaphus* *Rupicapra rupicapra* *Sus scrofa*	*Capreolus capreolus* *Cervus elaphus* *Dama dama* *Sus scrofa*	*Capreolus capreolus* *Cervus elaphus* *Rupicapra rupicapra* *Sus scrofa*
Medium-sized mammals	*Lepus europaeus* *Myocastor coypus*	*Lepus europaeus* *Oryctolagus cuniculus*	*Lepus europaeus* *Lepus timidus* *Marmota marmota*
Small mammals	*Erinaceus europaeus; Talpa europaea; Sciurus vulgaris;* Sorcidae; Gliridae; Microtidae; Muridae		

### Data collection

We monitored golden jackal and red fox populations from March 2017 to November 2018; in particular, we carried out seasonal monitoring sessions (spring: March–May; summer: June–August; autumn: September–November; winter: December–February) during a first study period from March 2017 to February 2018 in each study area, whereas we carried out monthly monitoring sessions during a second study period from June to November 2018 in the Goritian Karst study area.

We based data collection mainly on the recording of species indirect signs of presence. We adopted a Tessellation Stratified Sampling method [42, 43] subdividing each study area into 10 sample squares of 25 km^2^ (5 × 5 km) and randomly selecting three routes, among the existing foot-paths and dirt roads, within each square. During each monitoring session, we walked the selected routes to record species indirect signs of presence mainly corresponding to scats, footprints, and vocalizations; every sign of presence was autonomously evaluated by the researchers conducting the fieldwork and discordant or dubious records were discarded (Additional file 1: S1). We georeferred each sign of presence and recorded the type of vegetation where it was found. Scats were collected in polyethylene bags and stored for subsequent diet analyses.

Besides the collection of signs of presence, we carried out camera trapping sessions to increase species detection. Thus, during each sampling session, we settled 10–13 camera traps (n = 8 MULTIPIR 12 HD; n = 2 IR-PLUS BF 110°; n = 5 Scout Guard SG520) in opportunistic sites, mainly located along foot-paths and dirt roads, for a minimum period of 5 days (min. sampling period per study area = 50 days during each sampling session). Camera traps were settled mainly on trees approximately 0.5–2.0 m above the ground and set to record time and date when triggered. We programmed cameras to record videos (60 s) during the 24 h with a minimum time delay between consecutive ones (0 s) [44].

### Data analysis

We performed data analyses subdividing the total amount of observations into two seasons: a warm season, lasting from March to August (i.e., spring and summer), and a cold season, lasting from September to February (i.e., autumn and winter). Consequently, we considered nine sampling sessions within each season.

#### Space

The co-occurrence between species was evaluated using presence/absence data at different spatial scales: (i) a large spatial scale corresponding to the sample squares and (ii) a small spatial scale corresponding to the walked routes. We used the Sørensen similarity index:

$${Ss}_{i,j}=\frac{{2a}_{ij}}{{2a}_{ij}+{b}_{ij}+{c}_{ij}}$$

where *a*_ij_ represents the number of sampled sites with the simultaneous presence of two species *i* and *j*, and *b*_ij_ and *c*_ij_ are the number of sampled sites with the presence of only one species. This index varies between 0 (maximum segregation) and 1 (maximum co-occurrence, i.e., both considered species are present in all sampled sites) [45].

Moreover, we delineated species utilization distributions non-parametrically through a probability density function using the kernel method [46]. We used a fixed estimator and the smoothing parameter selected by the process of least squares cross validation (LSCV) [47, 48] to obtain narrow kernels useful to reveal small-scale details of the data structure [49]. Consequently, we measured utilization distributions overlap through the Utilization Distribution Overlap Index (UDOI):

$$UDOI = ~A_{{1,2}} \mathop {\iint }\limits_{{ - \infty }}^{\infty } UD_{1} \left( {x,y} \right)~ \times ~UD_{2} ~\left( {x,y} \right)~dxdy$$

which equals 0 for two ranges that do not overlap and equals 1 for two ranges that have complete overlap; values < 1 indicate less overlap relative to uniform space use, whereas values > 1 indicate that ranges have a non-uniform distribution and a high degree of overlap [50]. The analyses were performed using “adehabitatHR” package for R software [51].

#### Habitat

Seven land cover variables were obtained from the habitat map of the Friuli–Venezia Giulia region (http://irdat.regione.fvg.it/WebGIS/): urban areas, intensively cultivated lands (mainly cereals and legumes), permanent crops (vineyards and fruit orchards), extensively cultivated lands (also including small cultivated land patches with different cultivation types and crops interspersed with natural or semi-natural areas), pastures and grasslands, shrublands, woodlands (broad-leaved and coniferous woodlands). Ecological Niche Factor Analysis (ENFA) [52, 53] was carried out using all seasonal occurrence points against the background habitats, expressed as relative percentages of the different land cover variables calculated within sample squares of 2.5 × 2.5 km. ENFA summarizes the variables into uncorrelated factors and extracts two measures of a species realized niche along two axes: the marginality (M), which describes how far the species optimum is from the average environmental conditions, and the specialization (S), which is an indication of niche breadth relative to the environmental background. M generally ranges from 0 to 1, although the value can exceed one; values > 1 indicate that the niche deviates more relative to the habitat background composition, in other words, the species has specific habitat preferences compared to the available environment. S ranges from 1 to infinite; values > 1 indicates some forms of niche specialization, in other words, a decreasing niche breadth. To facilitate comparisons and easily interpret niche breadth, we also calculated the tolerance (T) as the inverse of S [52]. ENFA was calculated using “CENFA” package for R software [54].

#### Time

We estimated activity patterns non-parametrically through a probability density function using the kernel method [55] analysing data obtained by camera trap sampling. We considered as events only videos of the same species spaced 30 min to ensure capture independence [56–59]. We tested distribution uniformity using Watson’s test (U^2^) [60]. We performed pairwise comparisons between golden jackal and red fox activity patterns by estimating the coefficient of overlap (Δ) [55, 61]. We considered Δ_1_ estimator as the smaller sample had less than 75 records in both seasons [62]. To test for the reliability of the index and obtain 95% confidence intervals, we performed a smoothed bootstrap generating 1000 resamples [62]. Then we compared seasonal golden jackal and red fox distributions through Watson’s two-sample test (two-sample U^2^) to test for common distribution [60]. The activity pattern analyses were performed using “circular” and “overlap” packages for R software [62, 63].

#### Diet

Golden jackal and red fox diets were studied through scat analysis. We stored scats at − 20 °C for 30 days, then we analysed them to identify the consumed items from undigested remains: hairs, feathers, skulls, claws, and seeds. Each remain was identified by the comparison to a reference collection and atlas [64–67]. To describe the diet composition, the identified remains were grouped into nine food categories: (1) wild ungulates, (2) small mammals, (3) medium-sized mammals, (4) birds, (5) reptiles, (6) invertebrates, (7) fruit, (8) grasses, and (9) garbage. We estimated the proportion of consumed items for each scat and then we linked it to a percent volumetric class [68]. We assessed the adequacy of sample size with the Brillouin index (Hb; Additional file 1: S3).

We tested for significance of seasonal variations of the consumed categories by nonparametric multivariate analysis of variance (NPMANOVA) [69]. Moreover, we verified seasonal variations within categories by nonparametric analysis of variance (Kruskal–Wallis test). We evaluated the trophic niche overlap between golden jackal and red fox through Pianka’s index:

$${O}_{vs}=\frac{\sum {p}_{iv}{p}_{is}}{\sqrt{\sum }{{p}^{2}}_{iv}\sum {{p}^{2}}_{is}}$$

where *p*_ij_ is the proportion of the resource *i* out of the total resources used by the golden jackal, while *p*_if_ is the proportion of the resource *i* out of the total resources used by the red fox, and *i* could range from 1 to *n*, where *n* is the total number of food items considered. The value of index ranges from 0 (no overlap) to 1 (full overlap) [70]. To estimate the confidence intervals at 95% of the index distribution, we resampled the data 1000 times by the bootstrap method. Moreover, we tested for significance of specific variations of the consumed categories between seasons by two-way nonparametric multivariate analysis of variance (NPMANOVA) [69] and within categories by nonparametric analysis of variance (Kruskal–Wallis test). Diet analyses were performed using R software and specific packages as “stats” [71] and “npmv” [72].

## Results

### Collected data

During the study period we collected 277 records of golden jackal and 511 records of red fox presence. Considering the first study period (March 2017–February 2018), most of the records were collected in Goritian Karst study area (n = 105), while a few were collected in Tagliamento Valley study area (n = 18); no record was collected in Magredi study area (Table 2 and Additional file 1: Fig. 1 in S2). Conversely, the red fox was detected in every study area (Goritian Karst n = 153; Magredi n = 90; Tagliamento Valley n = 151) (Table 3 and Additional file 1: Fig. 2 in S2). Besides target species, in Magredi study area we recorded a few data of wolf presence (n = 6) (Additional file 1: Fig. 3 in S2).

**Table 2:** Records of golden jackal presence collected in Friuli–Venezia Giulia region from March 2017 to November 2018

Sampling period	Sampling season	Study area	Direct observations	Scats	Camera-trapping events	Footprints	Vocalizations	Carcasses
March 2017–February 2018	Warm season	Goritian Karst		24	2		8	
		Magredi						
		Tagliamento Valley		3	3		2	
	Cold season	Goritian Karst	2	10	44	8	7	
		Magredi						
		Tagliamento Valley		1	7	2		
June 2018–November 2018	Warm season			4	29	3	4	
	Cold season			11	93	2	8	
		Total	2	53	178	15	29

**Table 3:** Records of red fox presence collected in Friuli–Venezia Giulia region from March 2017 to November 2018

Sampling period	Sampling season	Study area	Direct observations	Scats	Camera-trapping events	Footprints	Vocalizations	Carcasses
March 2017–February 2018	Warm season	Goritian Karst		36	14	14	2	1
		Magredi		2	4	2		
		Tagliamento Valley	2	4	59	7		
	Cold season	Goritian Karst		11	3	72		
		Magredi		2	22	55	2	1
		Tagliamento Valley		4	10	65		
June 2018–November 2018	Warm season	Goritian Karst		16	11	22		
	Cold season			19	21	28		
		Total	2	94	144	265	4	2

Taking into consideration the aim of the research, we decided to exclude from the analyses red fox data collected in Magredi study area.

### Space

Co-occurrence between the golden jackal and the red fox was overall limited both at large spatial scale (warm season: Ss = 0.22; cold season: Ss = 0.13) and at small spatial scale (warm season: Ss = 0.07; cold season: Ss = 0.07).

**Fig. 2:**
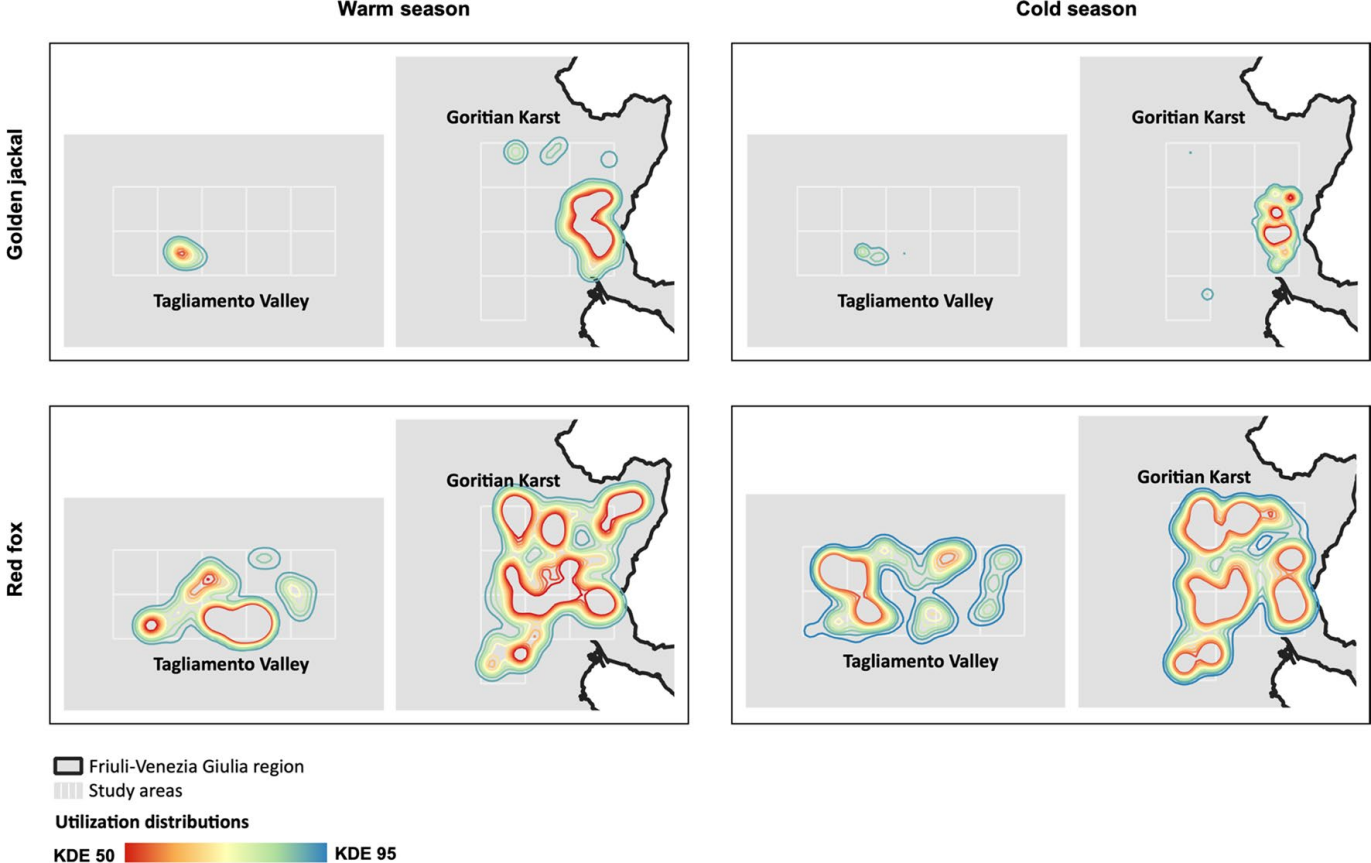
Utilization distributions of the golden jackal and the red fox in Friuli–Venezia Giulia region (March 2017–November 2018)

Kernel analysis revealed that golden jackal utilization distributions were restricted to a few sample squares of the study areas, whereas red fox utilization distributions were widespread and covered most of the study areas (Fig. 2). The estimated utilization distributions showed considerable variation in size between the two seasons both for the golden jackal (warm season: KDE95 [Kernel Density Estimate at 95%] = 92.9 km^2^; cold season: KDE95 = 44.9 km^2^) and the red fox (warm season: KDE95 = 388.9 km^2^; cold season: KDE95 = 442.1 km^2^). Overall, the spatial overlap between the golden jackal and the red fox was low during both seasons (warm season: UDOI = 0.20; cold season: UDOI = 0.17).

### Habitat

**Fig. 3:**
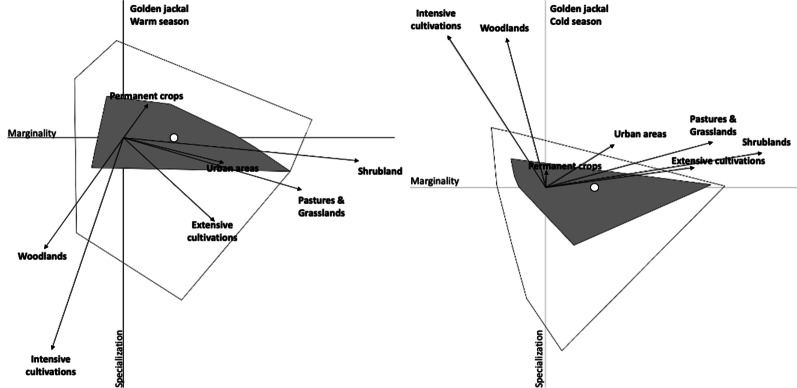
Ecological Niche Factor Analysis (ENFA) for suitable habitat for the golden jackal in Friuli–Venezia Giulia region (March 2017–November 2018). X-axis corresponds to the marginality axis; Y-axis corresponds to the first axis of specialization. Arrow length indicates the magnitude with which each variable accounts for the variance on each of the two axes. The white and grey areas correspond to the minimum convex polygon enclosing all the projections of the available and used points, respectively. White circle indicates niche position (median marginality) relative to the average background environment (the plot origin)

**Fig. 4:**
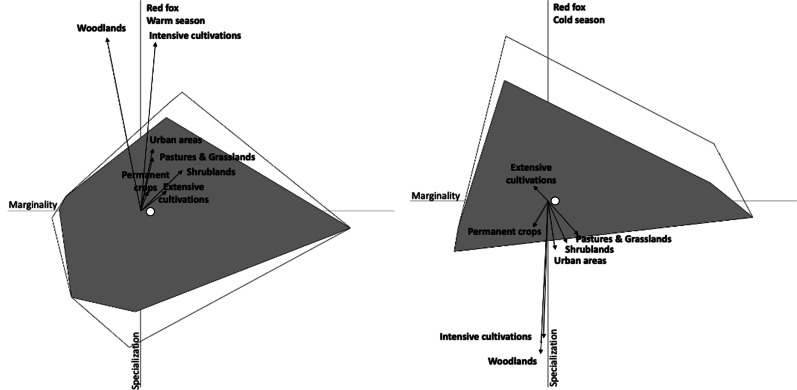
Ecological Niche Factor Analysis (ENFA) for suitable habitat for the red fox in Friuli–Venezia Giulia region (March 2017–November 2018). X-axis corresponds to the marginality axis; Y-axis corresponds to the first axis of specialization. Arrow length indicates the magnitude with which each variable accounts for the variance on each of the two axes. The white and grey areas correspond to the minimum convex polygon enclosing all the projections of the available and used points, respectively. White circle indicates niche position (median marginality) relative to the average background environment (the plot origin)

We considered 141 presence points (warm season: n = 63; cold season: n = 78) for the golden jackal and 329 presence points (warm season: n = 113; cold season: n = 216) for the red fox. The habitat of the golden jackal was rather different from the mean environmental conditions available within the study area, with the ENFA marginality factor slightly above the available background environment (warm season: M = 1.43; cold season: M = 1.42). The species had a narrow habitat niche breadth indicating highly specialized environmental requirements (warm season: S = 1.22 and T = 0.82; cold season: S = 1.14 and T = 0.88). Four and three significant ENFA factors explained 85.7% and 81.5% of the total variance respectively during warm and cold season (Table 4). The ENFA results indicated that the presence of the golden jackal was mainly linked to shrublands and pastures and grasslands during the warm season, while it was mainly linked to shrublands, extensively cultivated lands and pastures and grasslands during the cold season, as these variables had the highest coefficients on the marginality axis (Table 4 and Fig. 3). The habitat of the red fox did not deviate substantially from the mean environmental conditions available within the study areas, with the ENFA marginality below the available background environment (warm season: M = 0.21; cold season: M = 0.14). Obtained values indicated a negligible tendency of niche specialization (warm season: S = 1.02 and T = 0.98; cold season: S = 0.98 and T = 1.02). Accordingly, the coefficients on the marginality axis were rather low. Four significant ENFA factors explained 68.9% and 65.6% of the total variance respectively during warm and cold season (Table 5 and Fig. 4).

**Table 4:** Variance explained by the most significant factors (Marg = Marginality; Spec = Specialization) in the Ecological Niche Factor Analysis (ENFA) for suitable habitat for the golden jackal in Friuli–Venezia Giulia region (March 2017–November 2018)

	Warm season				Cold season		
ENFA axis	Marg	Spec1	Spec2	Spec3	Marg	Spec1	Spec2
Variance explained (%)	3.69	43.07	24.47	14.46	4.63	53.96	22.94
Predictors							
Shrublands	0.98	− 0.09	− 0.14	− 0.16	0.92	0.15	− 0.15
Woodlands	− 0.33	− 0.42	− 0.70	− 0.67	− 0.17	0.66	− 0.71
Intensively cultivated lands	− 0.30	− 0.81	− 0.63	− 0.66	− 0.42	0.67	− 0.61
Extensively cultivated lands	0.37	− 0.32	− 0.15	− 0.07	0.63	0.09	− 0.06
Permanent crops	0.10	0.12	− 0.09	− 0.07	0.00	0.07	− 0.08
Pastures and grasslands	0.74	− 0.20	− 0.19	− 0.20	0.71	0.20	− 0.20
Urban areas	0.41	− 0.09	− 0.18	− 0.20	0.29	0.19	− 0.20

**Table 5:** Variance explained by the most significant factors (Marg = Marginality; Spec = Specialization) in an Ecological Niche Factor Analysis (ENFA) for suitable habitat for the red fox in Friuli–Venezia Giulia region (March 2017–November 2018)

	Warm season				Cold season			
ENFA axis	Marg	Spec1	Spec2	Spec3	Marg	Spec1	Spec2	Spec3
Variance explained (%)	11.61	26.81	17.08	13.44	12.46	22.83	16.55	13.75
Predictors								
Shrublands	0.13	0.15	− 0.35	0.12	0.06	− 0.18	0.14	0.15
Woodlands	− 0.11	0.67	− 0.47	0.65	− 0.02	− 0.70	0.61	0.67
Intensively cultivated lands	0.05	0.65	− 0.57	0.68	− 0.02	− 0.63	0.70	0.65
Extensively cultivated lands	0.08	0.07	0.49	0.11	− 0.04	0.06	0.10	0.08
Permanent crops	0.02	0.07	− 0.13	0.12	− 0.05	− 0.11	0.11	0.05
Pastures and grasslands	0.04	0.21	− 0.20	0.20	0.10	− 0.15	0.24	0.20
Urban areas	0.04	0.24	− 0.19	0.15	0.02	− 0.22	0.20	0.22

### Time

**Fig. 5:**
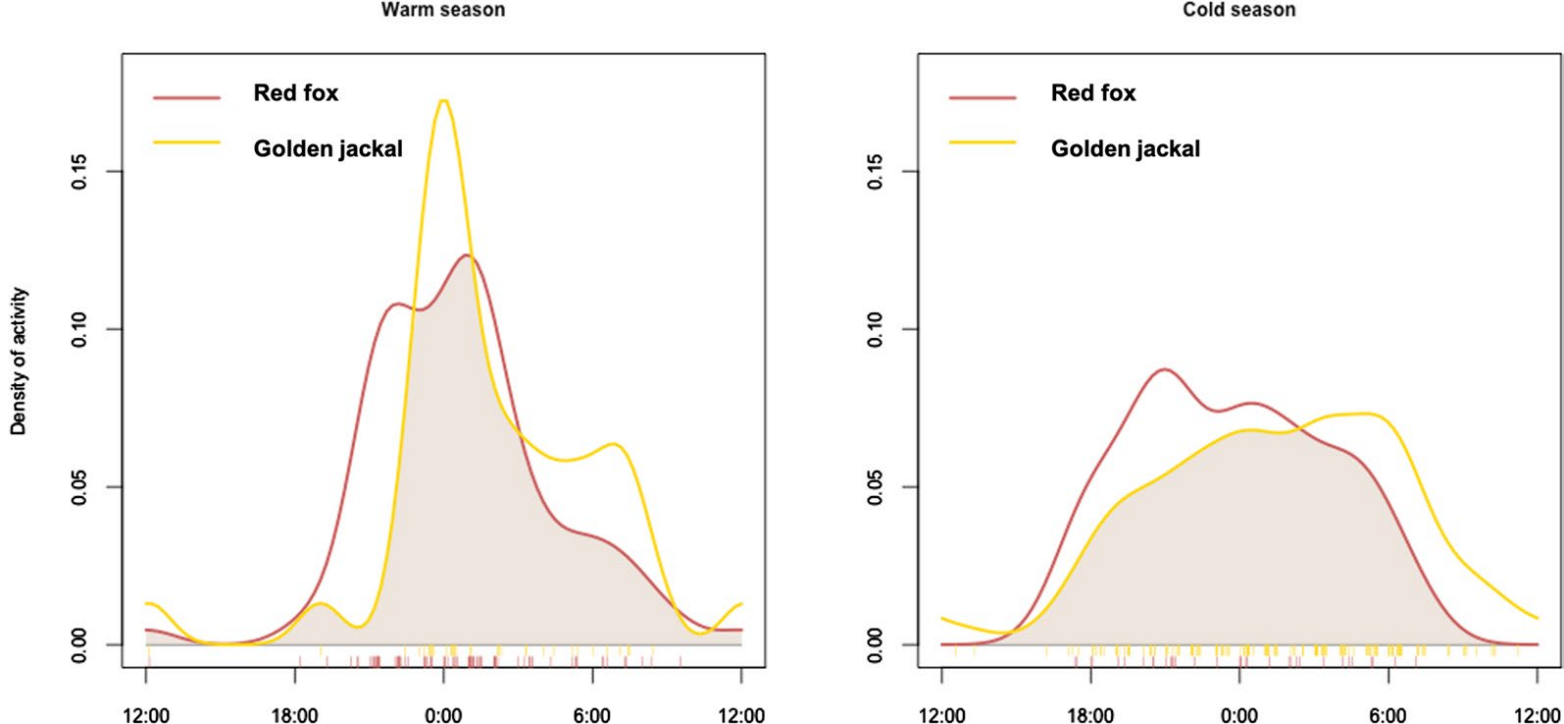
Activity patterns of the golden jackal and the red fox and interspecific temporal overlap in Friuli–Venezia Giulia region (March 2017–November 2018)

During 1134 trapping days (warm season: n = 585; cold season: n = 549), we collected 178 videos recording golden jackal activities (warm season: n = 34; cold season: n = 144) and 118 videos recording red fox activities (warm season: n = 84; cold season: n = 34). Both species had non-uniform patterns of activity during the 24 h being active especially at night and at dawn and dusk. In particular, during the warm season both species showed a marked activity peak around midnight and a less pronounced activity peak at 06:00; during the cold season both species had a prolonged active bout between dusk and dawn, but their main activity peaks diverged with the golden jackal activity peak at dawn (around 06:00) and the red fox activity peak around 21:00 (Table 6 and Fig. 5). Nevertheless, temporal overlap between the golden jackal and the red fox was extensive during both seasons, as the coefficient of overlap was close to 1 (warm season: Δ_1_ = 0.77; cold season: Δ_1_ = 0.82), with no significant difference between activity patterns of the two species (Table 6).

**Table 6:** Activity patterns of the golden jackal and the red fox in Friuli–Venezia Giulia region (March 2017–November 2018): non-uniformity of distributions and species temporal overlap

Season	Species	Single distributions		Distribution overlap		
		U^2^	P	Δ_1_ (CI)	Two-sample U^2^	P
Warm season	Golden jackal	0.84	< 0.01	0.77 (0.65–0.88)	0.13	> 0.10
	Red fox	2.11	< 0.01			
Cold season	Golden jackal	1.45	< 0.01	0.82 (0.69–0.93)	0.10	> 0.10
	Red fox	0.57	< 0.01

**Fig. 6:**
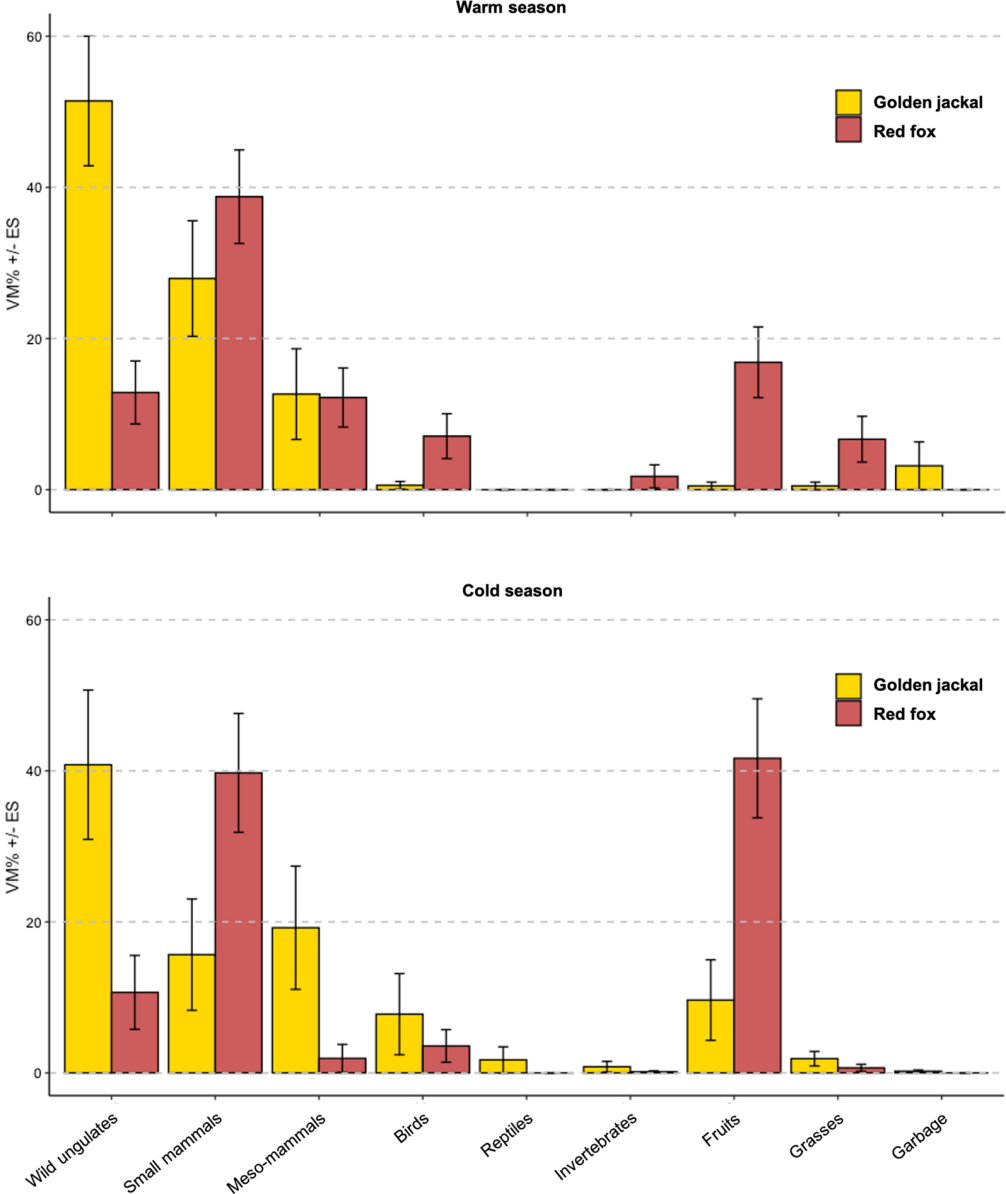
Food habits of the golden jackal and the red fox in Friuli–Venezia Giulia region (March 2017–November 2018): mean percent volume (VM% ± SE) of consumed categories

### Diet

We analysed 53 golden jackal scats (warm season: n = 31; cold season: n = 22) and 90 red fox scats (warm season: n = 56; cold season: n = 34). The sample size was adequate to represent both the golden jackal and the red fox diet (Additional file 1: Fig. 1 in S3). Scat analyses detected nine food categories consumed by the golden jackal and seven food categories consumed by the red fox (Fig. 6 and Additional file 1: Table 1 in S4). The most important consumed categories by the golden jackal were wild ungulates, followed by small mammals and medium-sized mammals; other seasonal important categories were birds and fruits (Fig. 6 and Additional file 1: Table 1 in S4). Golden jackal diet was significantly different between the seasons (NPMANOVA: F = 2.26; p = 0.024); in particular, the consumption of fruits (Kruskal–Wallis test: H = 7.91; df = 1; p = 0.005) and grasses (H = 7.59; df = 1; p = 0.006) was significantly higher during the cold season. The most important consumed categories by the red fox were small mammals, followed by fruits and wild ungulates (Fig. 6 and Additional file 1: Table 1 in S4). Even red fox diet was significantly different between the seasons (F = 2.76; p = 0.028), as the consumption of fruits was higher during the cold season (H = 8.89; df = 1; p = 0.003). Diet overlap between the two canids was medium–high during warm season (Pianka’s index: O = 0.68; CI = 0.42–0.99) and cold season (O = 0.53; CI = 0.35–0.75) without seasonal significant difference. Nevertheless, golden jackal and red fox diets were significantly different between seasons (F = 4.98; p < 0.0001; warm season: p = 0.004; cold season: p = 0.002). During warm season the consumption of wild ungulates was significantly higher for the golden jackal (H = 13.80; df = 1; p = 0.001), whereas the consumption of invertebrates (H = 4.16; df = 1; p = 0.041), fruits (H = 8.92; df = 1; p = 0.003) and grasses (H = 5.04; df = 1; p = 0.025) was significantly higher for the red fox. Similarly, during cold season the consumption of wild ungulates (H = 6.03; df = 1; p = 0.014) and medium-sized mammals (H = 5.37; df = 1; p = 0.020) was significantly higher for the golden jackal, whereas the consumption of small mammals (H = 5.57; df = 1; p = 0.018) and fruits (H = 7.68; df = 1; p = 0.005) was significantly higher for the red fox (Fig. 6).

## Discussion

Even though we choose our three study areas based on the most recent known distribution range of the golden jackal to carry out our research, we found no evidence of golden jackal presence in Magredi study area during our first study period; instead, interestingly we found evidence of wolf presence. In particular, the stable presence of a pair of wolves was documented throughout 2017, while the first event of reproduction was confirmed in 2018 [73]. The non-detection or the displacement of the golden jackal in newly established wolf ranges, due to some top-down effect induced by wolves on golden jackals, have been documented several times in Europe [74] and support the hypothesis that interspecific interactions between these large- and meso-predator may be similar to those observed in North America between the wolf and the coyote [75, 76].

The obtained results suggest marked spatial partitioning between the golden jackal and the red fox. Spatial segregation is one of the key mechanisms regulating coexistence within carnivore guild: species are sympatric across their range, but inverse relationships may be observed at local scales due to interspecific competition [20]. Accordingly, we observed not only a decreasing spatial overlap from large spatial scale to small spatial scale between the two species, but also a spatial displacement, considering the estimated utilization distributions, between species core areas: in other words, most frequented areas by the golden jackal overlapped less frequented areas by the red fox. Therefore, the observed spatial partitioning may represent the response of the subordinate species (i.e., the red fox) to the dominant species (i.e., the golden jackal) trying to reduce probabilities of direct encounters, namely a mechanism of dominant predator avoidance. Accordingly, spatial segregation between these two species has been documented elsewhere [33]. Interestingly Scheinin et al. [32] experimentally demonstrated that red foxes completely avoid direct encounters with golden jackals although the flight behaviour entails the abandonment of a very rich food patch. It is plausible that the extreme forms of interference competition, which are interspecific killing and intraguild predation [35, 77], may occur between these species similarly to what has been observed between coyotes and foxes ([8] and references therein). Evidence supporting this hypothesis may be the consumption of red foxes by golden jackals, which has been found in our study areas as well as in others [78].

Spatial partitioning is strongly related to habitat partitioning, another important mechanism promoting species coexistence. The golden jackal and the red fox are considered very adaptable species able to inhabit a wide range of habitats across the Eurasian continent [24, 26, 27, 29, 31]; however, our results suggest that the red fox may be noticeably more habitat generalist compared to the golden jackal. The main difference between species occurred in the magnitude of the marginality and specialization factors, where the golden jackal showed higher marginality and specialization than the red fox for most of the considered land cover variables. Indeed, the red fox occupied a broader habitat niche persisting in intensively cultivated areas and demonstrating higher tolerance to human-induced habitat modifications. The red fox occurred throughout the entire study areas, while the golden jackal was absent from approximately 50% of the red fox’s range (Additional file 1: S2). The golden jackal resulted to be specialist concerning its habitat niche: shrublands, natural open areas, such as pastures and grasslands, and extensively cultivated lands were positively associated with species presence. These kinds of habitats provide adequate resources, such as abundant and diverse prey, den and resting sites [41, 79]. Conversely, the intensive agricultural lands predominant in the plain zone of Goritian Karst study area unlikely can provide adequate resources for the golden jackal, because they are characterized by mono-specific fields of cereals and legumes that lead to a uniform landscape [41]. On the other hand, the red fox resulted to be generalist concerning its habitat niche: the species had no specific habitat preferences compared to the availability of the study areas. It is plausible that the presence of the habitat specialist, but dominant, species in less human-modified habitats might have led the habitat generalist, but subordinate, species to massively occupy intensively cultivated areas. In other words, the red fox behaved as subordinate but superior exploitative competitor and species adaptations to human-modified habitats may have enabled it to exploit areas unsuitable or suboptimal for the golden jackal [80].

Besides spatial and habitat partitioning, partitioning at the temporal scale may play a major role in relaxing competition between species with similar ecological requirements [59]. Conversely, we observed extensive temporal overlap along the diel cycle between the golden jackal and the red fox, both having predominant crepuscular and nocturnal activity patterns. Anyway, following the categories defined by Monterroso et al. [58], the two canids may be considered facultative nocturnal species, as they showed occasional activity events during the daytime. Very similar patterns have been observed also in other canids [56, 18, 81, 82] and may depend on either the physiological periodicity or the adaptation to the biological rhythms of their main prey species [22].

Being active at the same time but exploring different areas and habitats may lead sympatric species to infrequent encounters, thus it may represent an adequate mechanism to reduce potential competition [11, 83, 84].

Alternatively, the observed spatial and habitat partitioning may also be related to the partial diet overlap; in other words, the two species may use different areas for foraging. The golden jackal and the red fox are both generalist carnivores consuming a wide range of prey species across the Eurasian continent, from small mammals to wild ungulates; moreover, both species can alternatively behave as scavengers or active predators, even considering larger prey species [13, 85–87]. Our research showed that in Friuli–Venezia Giulia region the golden jackal and the red fox mainly shared three important prey categories, which were wild ungulates, small mammals, and medium-sized mammals. However, the obtained results also underline that, despite the substantial diet overlap, the two species might have occupied distinct trophic niches with the golden jackal mainly consuming wild ungulates and the red fox mainly consuming small mammals. The consumption of medium-sized mammals, the third shared food category, was of secondary importance for both canids. Generally, the food habits of both species were similar to those reported by previous researches, in particular those carried out in more natural and heterogeneous landscapes [88, 89]. Conversely in human-modified landscapes, e.g. intensive agricultural lands, the food habits of the two species tended to converge towards increased consumption of small mammals, mainly rodents [15, 16, 90]. Thus, in altered and highly uniform landscapes, where prey abundance and diversity may be limited, a larger dietary overlap between the golden jackal and the red fox is expected leading to a major potential competition for trophic resource. Our results on trophic differentiation could have been allowed by golden jackal and red fox specific predation behaviours. Specific hunting behaviours, with the golden jackal capable of cooperative hunting and therefore of preying on larger species [16], may have produced the observed diet partitioning. This may be particularly true considering the fact that the most consumed wild ungulate species by the golden jackal was the roe deer. This ungulate species may represent an easy prey for jackals because of the small body size (15–35 kg) and the hiding and solitary behaviour, in particular at birth time and during fawn lactation [13]. Nevertheless, predation cannot exclude scavenging: considering carnivores’ guild, canids are among the most avid scavengers [91]. Thus, as alternative explanation, the golden jackal may have excluded the red fox from wild ungulates’ carcasses achieving exclusive access to such foraging subsidies. According to most recent researches related to the suppression vs. facilitation hypothesis, because carcasses increase the likelihood of encountering a competitor (the “fatal attraction” hypothesis [92]), the red fox may avoid scavenging when other resources (e.g., small mammals) were abundant [91].

The observed patterns of resource use by the golden jackal and the red fox in our research were generally consistent with the predictions of the niche partitioning hypothesis, which is expected to favour interspecific coexistence [6]. We found a marked spatial and habitat partitioning between the two canids, but extensive temporal overlap along the diel cycle having both predominant crepuscular and nocturnal activity patterns. The analysis regarding habitat showed a high specialization of the golden jackal and a pronounced generalism of the red fox. Moreover, partial diet partitioning based on prey size resulted, with the golden jackal mainly feeding on wild ungulates and the red fox preferring small mammals. These results provided evidence that coexistence between the two canids was allowed by partial niche partitioning, despite the existing potential for competition [4]. Aggressive interactions between pairs of species are not constant and it is plausible that the interactions between the golden jackal and the red fox may vary from tolerance to predation, similarly to those observed between the coyote and the red fox [93].

## Conclusions

Based on the obtained results, we can deduce that the golden jackal and the red fox mainly segregated along the spatial, habitat, and trophic dimensions. This partitioning may be partially due to some ecological adaptations, i.e. the specialization in habitat use of the golden jackal vs. the superior exploitative ability in human-modified habitats of the red fox, and specific behaviours, i.e. alternative hunting behaviours (cooperative *vs.* solitary hunting), but it may be partially due also to the avoidance behaviour of the red fox aimed at reducing the competition with the golden jackal. Indeed, the observed spatial partitioning most likely reduced the probability of direct encounters between the two canids, in particular in more risky circumstances (e.g. scavenging on large prey carcasses) and may represent the response of the subordinate but superior exploitative species to relax interference competition with the dominant one.

This research contributes to our knowledge of interspecific interactions between potentially competing species providing useful new insights into the ecological and behavioural adaptability of the considered carnivore species. Such findings also provide basic knowledge on the ecology of a species, i.e. the golden jackal, hitherto poorly studied in Italy.

## Supplementary Information

Additional file 1: **S1.** Details on the sampling effort: collection of indirect signs of presence. **S2.** Details on collected data. **S3.** Diet analysis: adequacy of sample size. **S4.** Food habits of the golden jackal and the red fox.

## Data Availability

All data analysed during this study are included in the supplementary materials to this published article. The datasets used and analysed during the current study are available from the corresponding author on reasonable request.
