# Supplementary material for: Niche partitioning between sympatric wild canids: the case of the golden jackal (Canis aureus) and the red fox (Vulpes vulpes) in north-eastern Italy
Source: BMC Ecol Evol. 2021 Jun 22;21:129. doi: 10.1186/s12862-021-01860-3 (PMC8218446; doi:10.1186/s12862-021-01860-3)
Supplement: Supplementary file 1 — Additional file 1: S1. Details on the sampling effort: collection of indirect signs of presence. S2. Details on collected data. S3. Diet analysis: adequacy of sample size. S4. Food habits of the golden jackal and the red fox. [file 12862_2021_1860_MOESM1_ESM.pdf]

Niche partitioning between sympatric wild canids: the case of the golden jackal (*Canis aureus*) and the red fox (*Vulpes vulpes*) in north-eastern Italy

Additional material

S1. Details on the sampling effort: collection of indirect signs of presence …………………………… 2

S2. Details on collected data ……………………………………………………………………………………………. 5

S3. Diet analysis: adequacy of sample size ………………………………………………………………………. 8

S4. Food habits of the golden jackal and the red fox ………………………………………………………. 10

References ……. 12

S1. Details on the sampling effort: collection of indirect signs of presence

Table 1. Details regarding the sampling routes

Study area	N	Length			
		Mean (± SD)	Min.	Max.	Total
**Goritian Karst**	30	2434.68 ± 517.42 m	1753.50 m	4006.13 m	72.66 km
**Magredi**	30	2413.47 ± 441.42 m	1807.96 m	3477.28 m	72.40 km
**Tagliamento Valley**	30	2310.20 ± 399.90 m	1799.93 m	3891.49 m	69.31 km
**Total**	90				214.40 km

Figure 1. Location of the sampling routes (n = 90) within the three study areas

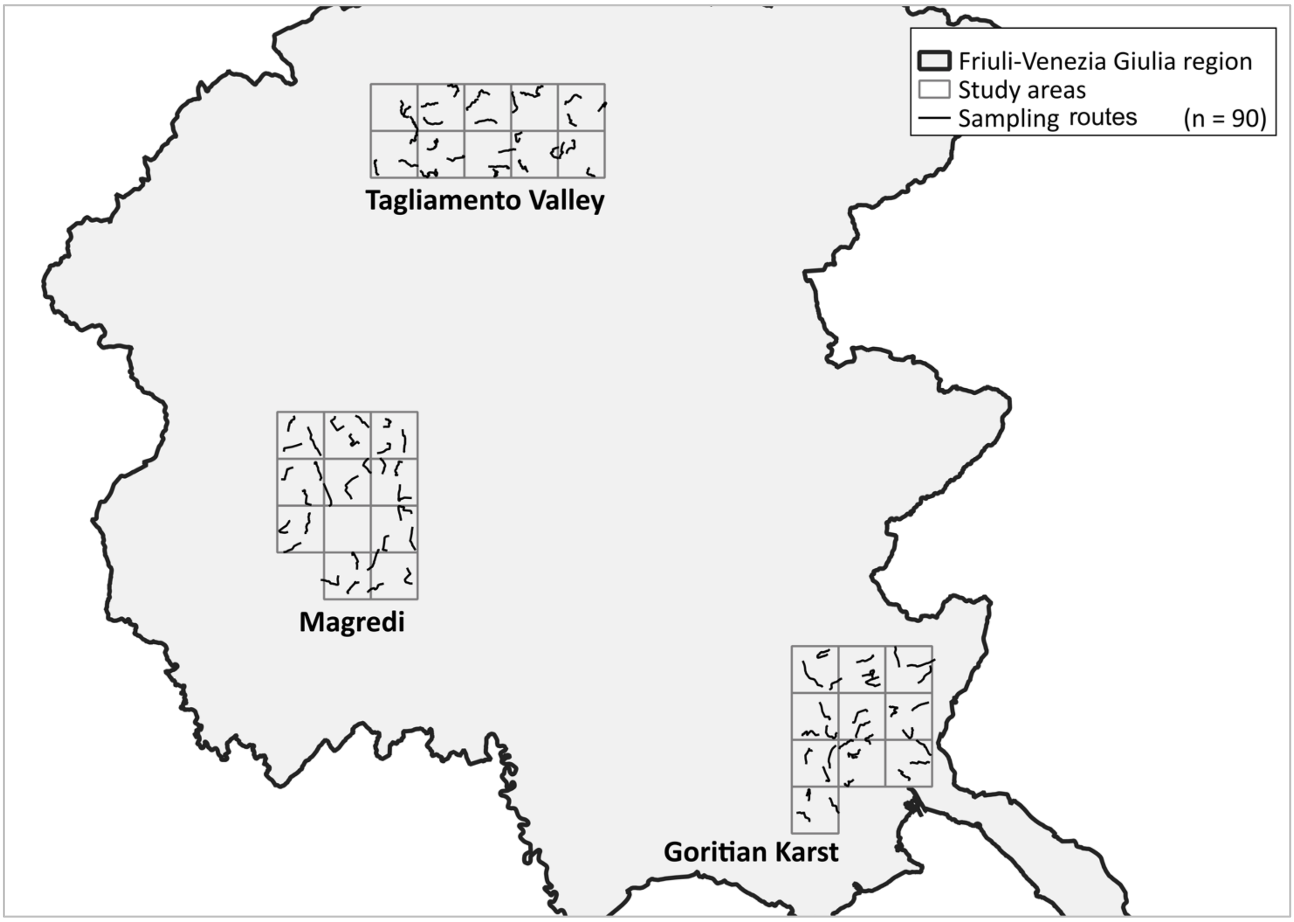

During each sampling session, at least three of us jointly walked the routes to record species signs of presence, mainly corresponding to scats, footprints, and vocalizations.

Scats were assigned to a species taking into consideration their size (both length and thickness), shape, odour, and location (Lanszki et al. 2006; Giannatos et al. 2010; Markov and Lanszki 2012); we didn't take into consideration their content. All scats collected during the first sampling period (March 2017 - February 2018) were photographed on the field and photos were stored for afterword controls (Figure 2): collected scats assigned to the red fox had a total length of 3-14 cm, while those assigned to the golden jackal had a total length of 13-38 cm; therefore, we excluded scats with a total length of 13-14 cm (Figure 3). We used these results to assign scats to a given species during the second sampling period (June 2018 - November 2018).

Footprints were recorded only if part of a track: pawprint shape and size, the stride and the respective positioning of the prints were considered for species assignment. Golden jackal pawprint is usually from 5 to 6 cm long and 3 to 4 cm wide; the two front digital pads are connected (not always visible) and claws are visible. Species trail has a footstep distance of 40-50 cm. Red fox pawprint is usually 5 cm long and 4 cm wide; it is characterized by a large space between the front two digital pads and the middle one. Species trail has a footstep distance of 30-40 cm (Bartol et al. 2019).

Scats and footprints with intermediate characteristics between the golden jackal and the red fox were discarded.

Golden jackal **vocalizations** correspond typically to high-pitched howling and yapping. Often multiple individuals vocalize concurrently. Howling can last up to several minutes (Bartol et al. 2019). Howls were assigned to the golden jackal only if the typical *yip-howl* sequences were heard (Hatlauf et al. 2016). Red fox vocalizations correspond typically to barks, a combination of a series of three to six sounds that change in a fast sequence and in some cases, it can even turn into screeching (Bartol et al. 2019).

Figure 2. Scats assigned to the golden jackal (on the left) and the red fox (on the right)

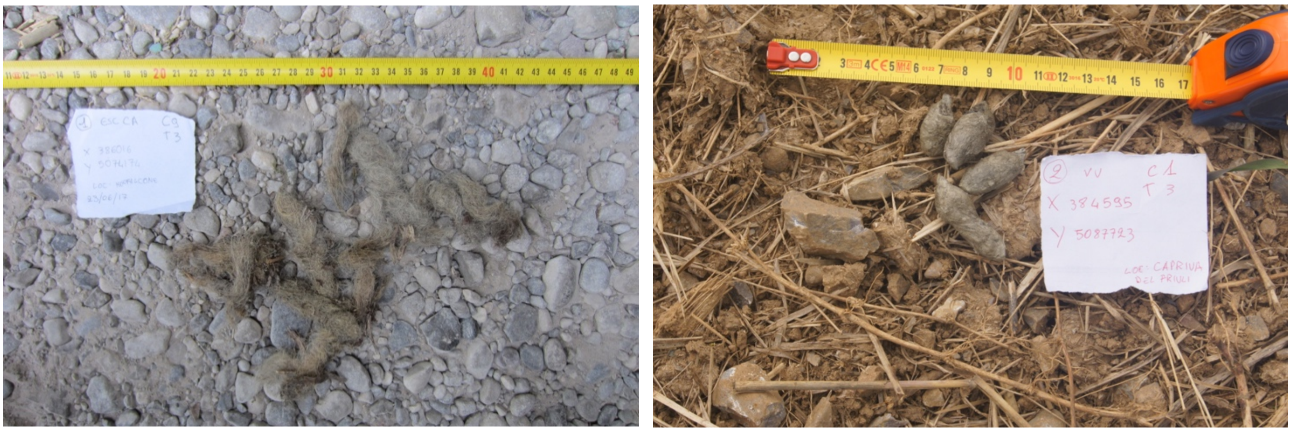

Figure 3. Total length of scats assigned to the target species: those assigned to the golden jackal had a mean (± SE) total length of 21.8 ± 1.5 cm, while those assigned to the red fox had a mean (± SE) total length of 8.8 ± 0.4 cm

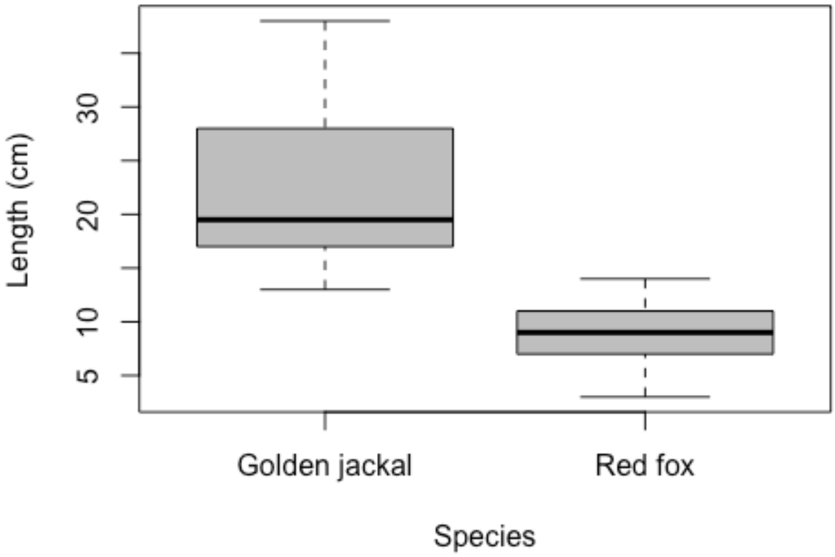

S2. Details on collected data

Figure 1. Golden jackal records collected in Friuli-Venezia Giulia region from March 2017 to November 2018

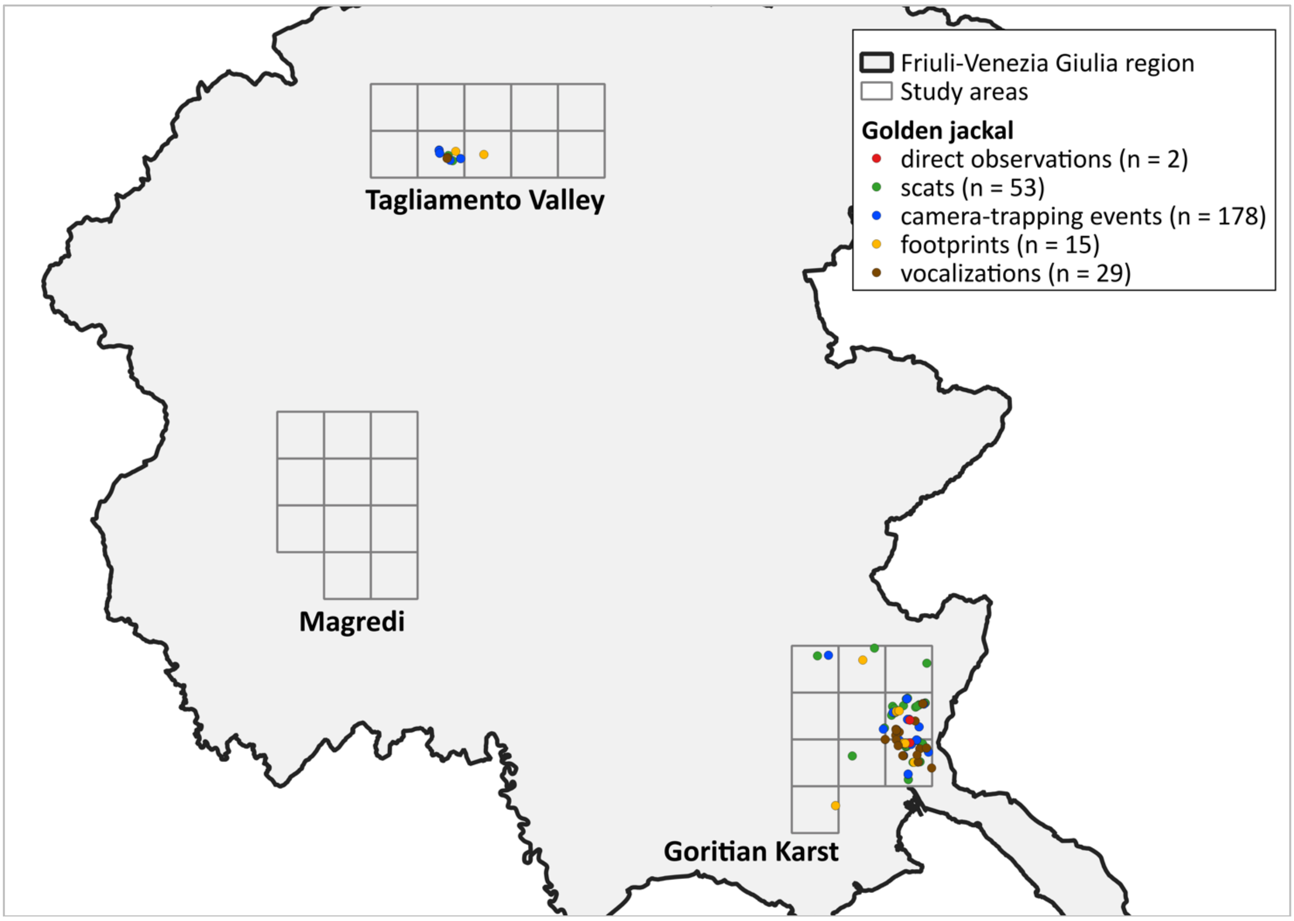

Figure 2. Red fox records collected in Friuli-Venezia Giulia region from March 2017 to November 2018

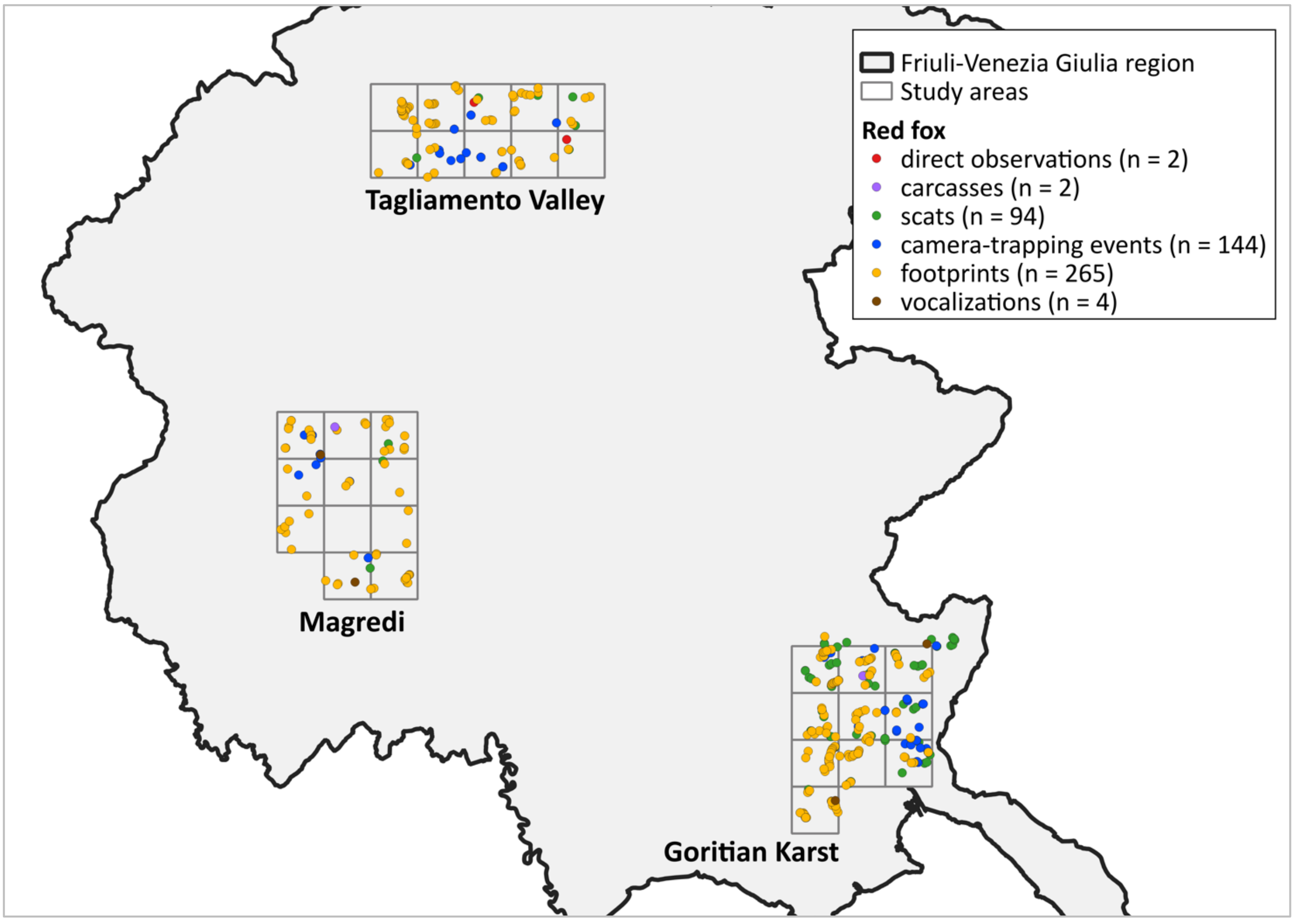

Figure 3. Wolf records collected in Friuli-Venezia Giulia region from March 2017 to February 2018

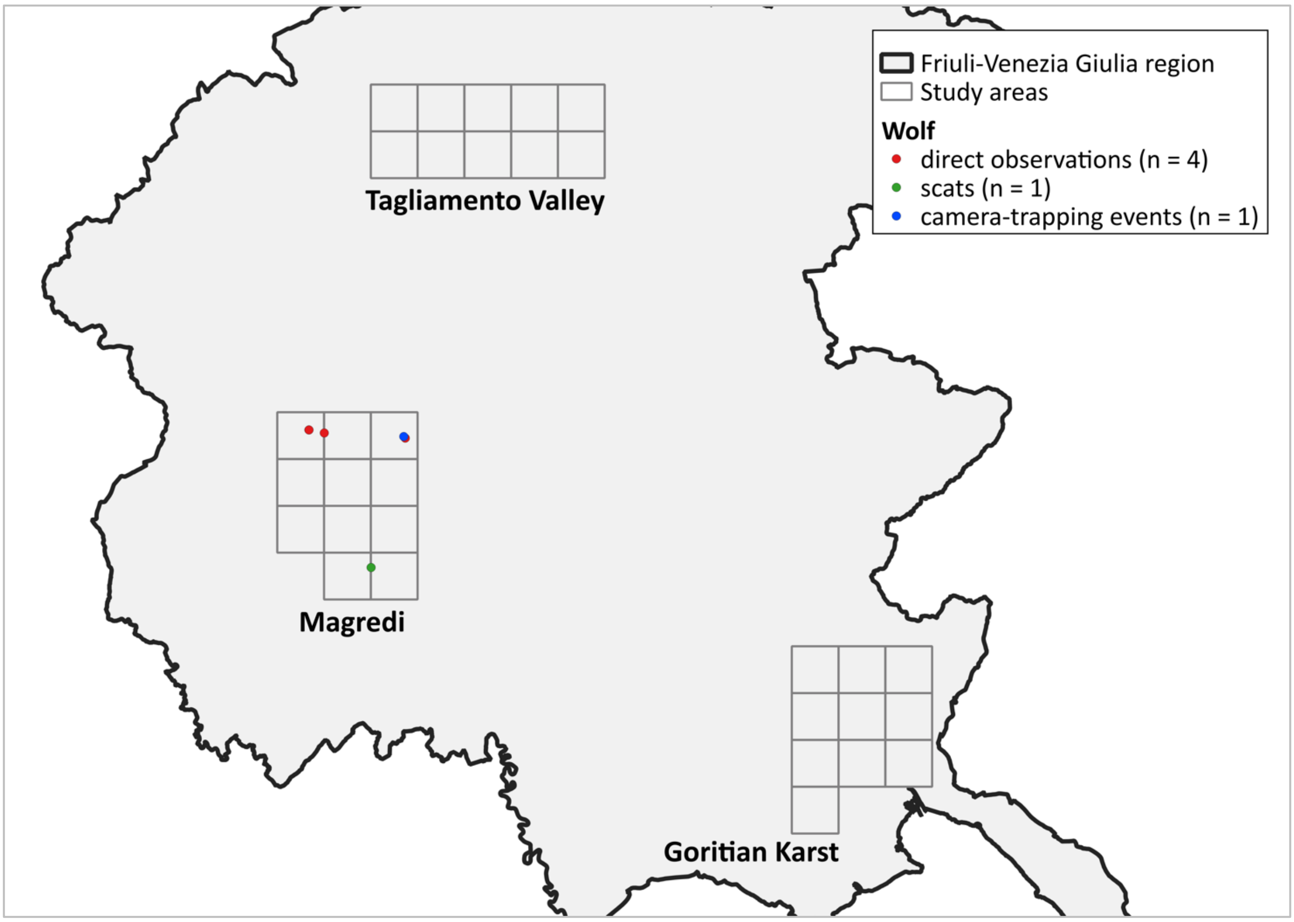

S3. Diet analysis: adequacy of sample size

Data analysis

We assessed the adequacy of sample size with the Brillouin index (Hb):

$$H_b = \frac{lnN! - \sum lnn_i}{N}$$

where Hb is the diversity of prey in the sample, N is the total number of individual prey taxa in all samples and ni is the number of individual prey taxa in the ith category. A diversity curve was then calculated by increments of two samples randomly taken. For each sample, a value of Hb was calculated and then resampled 1000 times by the bootstrap method to obtain the mean and 95% confidence interval. We determined the adequacy of the sample size by whether an asymptote was reached in the diversity curve and in another curve calculated from the incremental change in each Hb with the addition of two more samples. Both curves were plotted against the number of analysed scats (Glen et al. 2006; Hass 2009).

Results

The sample size was adequate to represent both the golden jackal and the red fox diet. The diversity curves reached the asymptote and the incremental change curves dropped below 1% with n < 14 scats for each species in each considered season (Figure 1).

Figure 1. Adequacy of the size of samples used to investigate golden jackal and red fox diet in Friuli-Venezia Giulia region (March 2017 - November 2018)

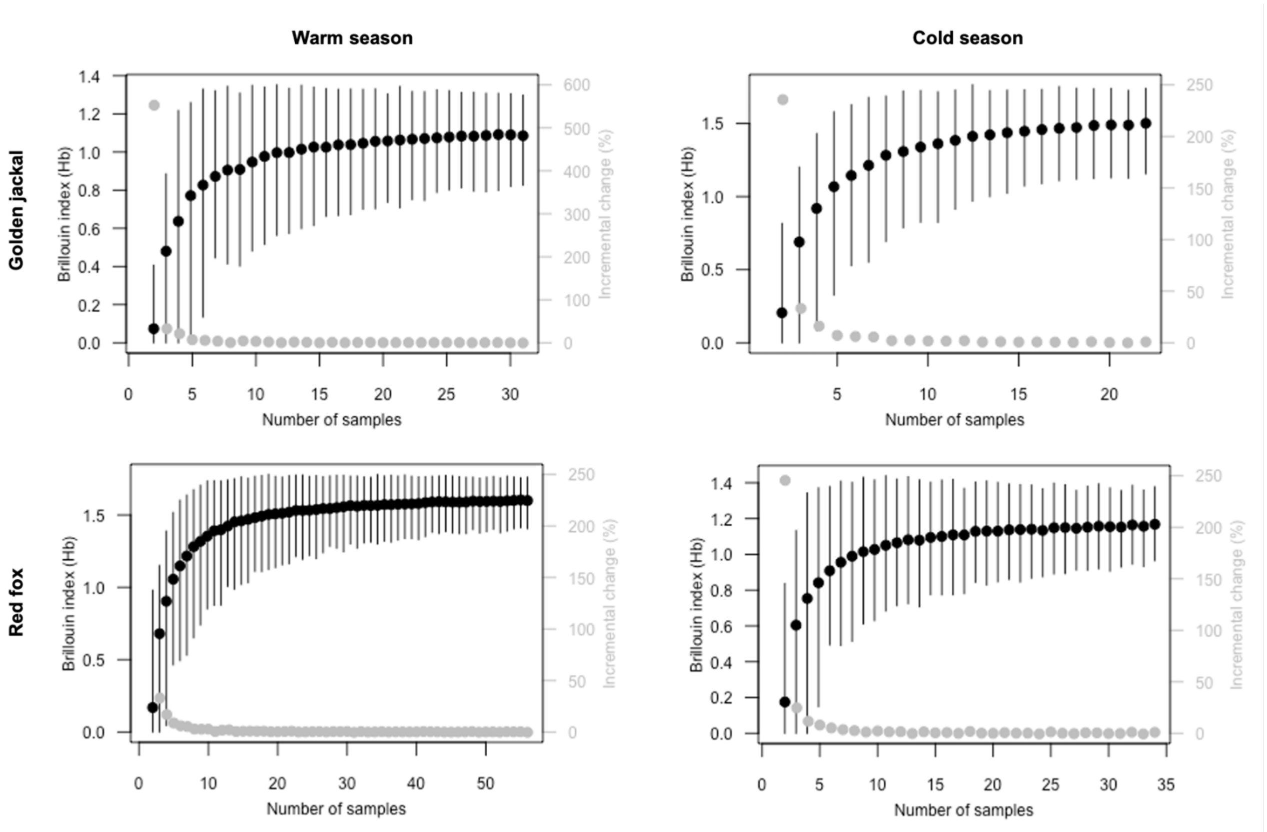

S4. Food habits of the golden jackal and the red fox

Table 1. Consumed categories and species by the golden jackal and the red fox in Friuli-Venezia Giulia region (March 2017 - November 2018)

(MV% = mean percent volume of food items in the diet)

Categories and species	Golden jackal (n = 53)				Red fox (n = 94)			
	Warm season (n = 31)		Cold season (n = 22)		Warm season (n = 58)		Cold season (n = 36)	
	MV%	SE	MV%	SE	MV%	SE	MV%	SE
**Wild ungulates**	**51.43**	**8.57**	**40.81**	**9.89**	**12.86**	**4.17**	**10.66**	**4.89**
Sus scrofa	23.35	7.46	7.43	5.17	6.88	3.08	5.86	3.78
Capreolus capreolus	28.08	7.64	33.38	9.67	5.98	2.92	4.81	3.38
**Small mammals**	**27.94**	**7.64**	**15.66**	**7.37**	**38.76**	**6.19**	**39.74**	**7.87**
Talpa sp.	3.16	3.16	0.00	0.00	1.75	1.75	0.00	0.00
Crocidura sp.	6.82	4.40	8.35	5.77	0.68	0.68	1.85	1.85
Sorex sp.	2.76	2.76	0.00	0.00	0.72	0.68	0.00	0.00
Apodemus sp.	0.00	0.00	2.86	2.86	8.58	3.49	0.00	0.00
Mus musculus	3.16	3.16	0.00	0.00	3.28	2.30	0.00	0.00
Rattus sp.	5.92	4.13	0.00	0.00	9.03	3.58	25.09	6.88
Microtus sp.	1.73	1.31	0.00	0.00	12.29	4.37	1.12	1.12
Myodes glareolus	0.00	0.00	0.00	0.00	0.00	0.00	2.88	2.88
Glis glis	0.00	0.00	4.45	4.45	0.68	0.68	2.96	2.88
Eliomys quercinus	1.23	1.23	0.00	0.00	0.00	0.00	0.00	0.00
Muscardinus avellanarius	0.00	0.00	0.00	0.00	1.75	1.75	2.96	2.88
Sciurus vulgaris	3.16	3.16	0.00	0.00	0.00	0.00	2.88	2.88
**Medium-sized mammals**	**12.65**	**6.00**	**19.23**	**8.16**	**12.19**	**3.91**	**1.93**	**1.85**
Lepus europaeus	0.00	0.00	10.32	6.13	6.44	2.85	1.93	1.85
Oryctolagus cuniculus	0.00	0.00	4.45	4.45	0.00	0.00	0.00	0.00
Vulpes vulpes	9.48	5.29	0.00	0.00	0.68	0.68	0.00	0.00
Meles meles	3.16	3.16	0.00	0.00	0.04	0.04	0.00	0.00
Mustela sp.	0.00	0.00	4.45	4.45	0.00	0.00	0.00	0.00
Martes sp.	0.00	0.00	0.00	0.00	5.03	2.86	0.00	0.00
**Birds**	**0.58**	**0.50**	**7.78**	**5.37**	**7.08**	**2.97**	**3.57**	**2.16**
Galliformes	0.00	0.00	3.89	3.89	2.03	1.77	0.00	0.00
Passeriformes	0.00	0.00	0.00	0.00	0.01	0.01	0.07	0.07
Columbiformes	0.00	0.00	0.00	0.00	0.68	0.68	0.00	0.00
Falconiformes	0.08	0.08	0.00	0.00	0.04	0.04	1.12	1.12
Piciformes	0.00	0.00	0.00	0.00	0.00	0.00	0.07	0.07

**Categories and species**	**Golden jackal** (n = 53)				**Red fox** (n = 94)			
	**Warm season** (n = 31)		**Cold season** (n = 22)		**Warm season** (n = 58)		**Cold season** (n = 36)	
	**MV%**	**SE**	**MV%**	**SE**	**MV%**	**SE**	**MV%**	**SE**
Indeterminate	0.50	0.50	3.89	3.89	2.57	1.68	2.31	1.89
**Reptiles (Indet.)**	**0.00**	**0.00**	**1.73**	**1.73**	**0.00**	**0.00**	**0.00**	**0.00**
**Invertebrates**	**0.00**	**0.00**	**0.82**	**0.71**	**1.76**	**1.53**	**0.16**	**0.10**
Orthoptera	0.00	0.00	0.11	0.11	0.00	0.00	0.00	0.00
Coleoptera	0.00	0.00	0.70	0.70	1.75	1.53	0.09	0.07
Indeterminate	0.00	0.00	0.00	0.00	0.01	0.01	0.07	0.07
**Fruits**	**0.50**	**0.50**	**9.65**	**5.33**	**16.86**	**4.68**	**41.66**	**7.88**
Vitis sp.	0.00	0.00	0.82	0.71	0.60	0.39	25.40	6.99
Prunus sp.	0.34	0.34	0.00	0.00	1.13	1.13	0.00	0.00
Corylus sp.	0.00	0.00	0.00	0.00	0.01	0.01	0.00	0.00
Morus sp.	0.00	0.00	0.00	0.00	0.60	0.39	0.00	0.00
Celtis sp.	0.00	0.00	8.60	5.36	0.00	0.00	13.38	5.69
Fragaria sp.	0.00	0.00	0.00	0.00	12.69	4.07	2.88	2.88
Cornus sp.	0.16	0.16	0.00	0.00	0.00	0.00	0.00	0.00
Quercus sp.	0.00	0.00	0.11	0.11	0.00	0.00	0.00	0.00
Indeterminate	0.00	0.00	0.11	0.11	1.84	1.75	0.00	0.00
**Grasses**	**0.50**	**0.50**	**1.89**	**0.96**	**6.68**	**3.03**	**0.68**	**0.47**
**Garbage**	**3.16**	**3.16**	**0.23**	**0.16**	**0.00**	**0.00**	**0.00**	**0.00**

References

Bartol M, Černe R, Krofel M, et al (2019) A fieldguide for investigating damages caused by carnivores brown bear, grey wolf, golden jackal, red fox, Eurasian lynx. Slovenia Forest Service, Ljubljana

Giannatos G, Karypidou A, Legakis A, Polymeni R (2010) Golden jackal (*Canis aureus* L.) diet in Southern Greece. Mamm Biol 75:227–232. https://doi.org/10.1016/j.mambio.2009.03.003

Glen AS, Dickman CR (2006) Diet of the spotted-tailed quoll (*Dasyurus maculatus*) in eastern Australia: effects of season, sex and size. J Zool 269:241–8

Hass CC (2009) Competition and coexistence in sympatric bobcats and pumas. J Zool 278:174–80

Hatlauf J, Banea OC, Lapini L (2016) Assessment of golden jackal species (*Canis aureus*, L. 1758) records in natural areas out of their known historic range. Technical Report, GOJAGE Criteria and Guidelines. Gojage E-Buletin, 12

Lanszki J, Heltai M, Szabó L (2006) Feeding habits and trophic niche overlap between sympatric golden jackal (*Canis aureus*) and red fox (*Vulpes vulpes*) in the Pannonian ecoregion (Hungary). Can J Zool 84:1647–1656. https://doi.org/10.1139/z06-147

Markov G, Lanszki J (2012) Diet composition of the golden jackal, *Canis aureus* in an agricultural environment. Folia Zool 61:44–48. https://doi.org/10.25225/fozo.v61.i1.a7.2012
